# Características de la población en rehabilitación cardiaca fase II de una clínica de IV nivel

**DOI:** 10.15446/rsap.V25n4.105237

**Published:** 2023-07-01

**Authors:** Alvaro Rodríguez-Lázaro, Andrés E. Hernández-Roa, Jenny P. Castillo-Gómez, Ana M. Rodríguez-Lozano

**Affiliations:** 1 AR: MD. Esp. Medicina Física y Rehabilitación. Clínica Universitaria Colombia. Bogotá, Colombia. alverodriguez@keralty.co Clínica Universitaria Colombia Medicina Física y Rehabilitación Clínica Universitaria Colombia Bogotá Colombia alverodriguez@keralty.co; 2 AH: MD. Esp. Medicina del Deporte. Clínica Universitaria Colombia. Bogotá, Colombia. aehernandez@keralty.co Clínica Universitaria Colombia Medicina del Deporte Clínica Universitaria Colombia Bogotá Colombia aehernandez@keralty.co; 3 JC: MD. Caja Colombiana de Compensación Colsubsidio. Bogotá, Colombia. jpaolacastillog@gmail.com Caja Colombiana de Compensación Colsubsidio Caja Colombiana de Compensación Colsubsidio Bogotá Colombia; 4 AR: MD. Esp. Medicina Física y Rehabilitación, Clínica Universitaria Colombia. Bogotá, Colombia. anamrodriguez@colsanitas.com Clínica Universitaria Colombia Medicina Física y Rehabilitación Clínica Universitaria Colombia Bogotá Colombia anamrodriguez@colsanitas.com

**Keywords:** Factores de riesgo, rehabilitación cardiaca, prevención secundaria *(fuente: DeCS, BIREME)*, Risk factors, cardiac rehabilitation, secondary prevention *(source: MeSH, NLM)*

## Abstract

**Objetivos:**

Caracterizar la población en fase II de rehabilitación cardiaca de la Clínica Universitaria Colombia, entre el 1.° de agosto del 2017 y el 31 de diciembre del 2019. Establecer la prevalencia de los factores de riesgo y los diez diagnósticos más frecuentes.

**Materiales y Métodos:**

Estudio cuantitativo descriptivo. Se analizó una base de datos de los pacientes que completaron 24 sesiones de ejercicio.

**Resultados:**

De 1 737 sujetos incluidos, el 61,5% fueron hombres. La edad promedio fue 63 años. El IMC promedio fue 26,7 kg/m^2^ (preobesidad). Los factores de riesgo prevalentes fueron: sedentarismo (71,2%), preobesidad-obesidad (71,2%), HTA (66,%), obesidad abdominal (60,5%), dislipidemia (56,9%), tabaquismo (42,8%) y diabetes (24,1%). El 70,5% de la población tuvo fracción de eyección conservada, el 11,8% fracción de eyección límite y el 13,9 % fracción de eyección reducida. En hombres predominó el sobrepeso, mientras que en mujeres lo hizo la obesidad grado I-II y abdominal.

**Conclusión:**

El sedentarismo y el estado de sobrepeso/obesidad son los principales factores de riesgo que afectan a nuestra población. Como medidas de control, nuestro programa debe fortalecer la orientación nutricional y promocionar el gasto calórico mediante el ejercicio físico.

## Abstract (en)

**Objectives:**

To characterize the population attending phase II of cardiac rehabilitation at the Colombia University Clinic, from August 1, 2017 to December 31, 2019. Establish the prevalence of risk factors and the ten most frequent diagnoses at admission.

**Materials and Methods:**

Quantitative descriptive study. A database of patients who completed 24 exercise sessions was systematically analyzed.

**Results:**

Of 1,737 subjects included, 61.5% were men. The average age was 63 years. The B.M.I. average was 26.7 kg/m^2^ (Preobesity). The prevalent risk factors were: Sedentary lifestyle (71.2%), Pre-obesity-obesity (71.2%), HBP (66.1%), Abdominal obesity (60.5%), Dyslipidemia (56.9%), Smoking (42.8%), Diabetes (24.1%). 70.5 % of the population had a preserved ejection fraction, 11.8 °% had a borderline ejection fraction, and 13.9% had a reduced ejection fraction. Overweight prevailed in men, while grade I - II and abdominal obesity prevailed in women.

**Conclusion:**

Sedentary lifestyle and overweight/obesity are the main risk factors affecting our population. Our program should strengthen nutritional guidance and promote caloric expenditure through physical exercise as control measures.

Las enfermedades cardiovasculares (ECV) lideran las causas de muerte y discapacidad global, lo cual eleva los costos de atención en salud. El 80 % de esta carga impacta en los países de bajos recursos, con un aumento reciente en su frecuencia 1,2.

En el 2016, las ECV en la región de las Américas (excepto Canadá), causaron 1,9 millones de muertes y representaron el 28,1% de las defunciones por enfermedades no transmisibles. La tasa de mortalidad fue de 150,7 por 100 000 habitantes y afectó más a hombres (185,2/100 000) que a las mujeres (121,6/100 000). En Colombia, las ECV contribuyeron al 29,7% de las muertes por enfermedades no transmisibles, con una tasa de defunción estandarizada por edad de 185,6 por 100 000 habitantes 3.

Un factor de riesgo cardiovascular (FRCV) es una característica biológica, condición o comportamiento que aumenta la probabilidad de enfermar o morir por ECV 4. Se clasifican tradicionalmente en "no modificables" (edad, raza, género, hereditarios) y "modificables" (hipertensión arterial, diabetes, sobrepeso/obesidad, dislipidemia, sedentarismo, alimentación no saludable, consumo de tabaco, entre otros). Estos últimos pueden corregirse si se adopta un estilo de vida saludable y siendo más adherentes al tratamiento.

Los FRCV "secundarios" abarcan estrés, hormonas sexuales, anticonceptivos orales y alcohol. Además, se postulan FRCV "no tradicionales" como estresores psicosociales, contaminación ambiental e inflamación, entre otros 1,2,5.

La globalización transformó las costumbres alimenticias en Colombia, se adoptaron hábitos poco saludables y se elevó el impacto nocivo de los FRCV a niveles alarmantes: en el 2014 la hiperglucemia afectó al 8,5%. La prevalencia de la hipertensión arterial (HTA) fue del 19,2% en el 2015. En el 2016, la tasa de mortalidad por diabetes mellitus tipo 2 (DMT2) fue de 21,3 por 100 000 habitantes, mientras que el sobrepeso/obesidad alcanzó el 59% y el sedentarismo el 44%. En el 2017, el tabaquismo tuvo una prevalencia del 8,2%. En el 2018, los adultos colombianos consumieron 5,7 L de alcohol puro por persona-año 3.

La rehabilitación cardiaca (RC) es una alternativa eficaz que integra estrategias educativas, psicosociales y de entrenamiento físico que impactan positivamente en los FRCV. Así, mejora la capacidad física, reduce la mortalidad hasta en un 32%, como también la rehospitalización, mediante adaptaciones fisiológicas, metabólicas y funcionales con el ejercicio 5-9. A pesar de avances en prevención y control, la falta de información actualizada sobre los FRCV podría aumentar su prevalencia y las ECV en el país 4.

Este trabajo se une a iniciativas globales para fortalecer estrategias de prevención de ECV basadas en el conocimiento de la población y sus FRCV, con influencia del programa de RC 1,10.

## OBJETIVOS

- Caracterizar a la población participante de la fase II del programa de RC de la Clínica Universitaria Colombia en Bogotá en el periodo de agosto 1 del 2017 a diciembre 31 del 2019.
- Describir las variables sociodemográficas y antropométricas de la población en estudio.
- Establecer la prevalencia de los FRCV y los 10 diagnósticos más frecuentes de ingreso al programa.

## METODOLOGÍA

### Diseño

Estudio cuantitativo descriptivo, de tipo de diseño de series de casos.

### Población de estudio

Pacientes inscritos en la fase II del programa de RC de la Clínica Universitaria Colombia en Bogotá, entre el 1.° de agosto del 2017 y el 31 de diciembre del 2019.

### Criterios de inclusión

Pacientes inscritos entre el 1." agosto del 2017 y el 31 de diciembre del 2019 que completaron 24 sesiones de fase II de RC, registrados en la base de datos.

### Criterios de exclusión

Pacientes que no completaron 24 sesiones en el tiempo estipulado.

### Selección de la muestra

Muestreo no probabilístico por conveniencia de todos los pacientes asistentes al programa durante el periodo de estudio.

### Conducción del estudio, fuentes y sistematización de la información

El servicio de RC atiende a pacientes de la IPS y a remitidos de otras instituciones. La fase II incluye educación en salud y 24 sesiones de ejercicio supervisadas por un equipo médico especializado. La estratificación del riesgo y la posibilidad de ingreso se determinan en la consulta.

Dos médicos fisiatras registraron datos en el *Software* de historia clínica y un formato físico, y así se estableció la matriz de variables del estudio (Tabla 1). Tras las 24 sesiones, el médico de RC ingresó la información en una base de datos de Excel®.

**Tabla 1:** Matriz de variables

Nombre	Definición operativa	Nivel	Naturaleza	Escala	Unidad
Edad	Años de vida	De razón	Cuantitativa	Continua	Años
Sexo	Según su género	Nominal	Cualitativa	No aplica	Femenino, masculino
Peso	Masa corporal medible	De razón	Cuantitativa	Continua	Kilogramos
Talla	Medida de altura corporal	De razón	Cuantitativa	Continua	Centímetros
Índice de masa corporal	Clasificación del estado ponderal	De razón	Cuantitativa	Continua	Kilogramos/metros al cuadrado
Perímetro abdominal	Medida de circunferencia abdominal	De razón	Cuantitativa	Continua	Centímetros
Fracción de eyección	Obtenido de estudios paraclínicos	De razón	Cuantitativa	Continua	Porcentaje
Estratificación del riesgo	Riesgo cardiovascular según AACVPR	Ordinal	Cualitativa	No aplica	Bajo, moderado, alto
Diagnóstico de ingreso	Enfermedad de remisión	Nominal	Cualitativa	No aplica	iam, revascularización miocárdica, etc.
Hipertensión arterial	Diagnóstico confirmado	Nominal	Cualitativa	No aplica	Sí, no
Diabetes	Diagnóstico confirmado	Nominal	Cualitativa	No aplica	Sí, no
Dislipidemia	Diagnóstico confirmado	Nominal	Cualitativa	No aplica	Sí, no
Tabaquismo	Consumo previo o actual	Nominal	Cualitativa	No aplica	Sí, no
Sobrepeso	IMC > o igual a 25	Nominal	Cualitativa	No aplica	Sí, no
Sedentarismo	Clasificación de actividad física	Nominal	Cualitativa	No aplica	Sí, no
Familiares	Antecedente de ecv en la familia	Nominal	Cualitativa	No aplica	Sí, no

Otro investigador verificó la calidad de la información, para lo cual controló el 5% de los datos seleccionados aleatoriamente y realizó una validación con el software Excel®.

### Análisis de la información

En la base de datos de Excel®, la variable *índice de masa corporal* se recodificó según la clasificación nutricional de la OMS 11. El *perímetro abdominal* se recodificó en obesidad abdominal, según la guía de práctica clínica de obesidad del Ministerio de Salud de Colombia 12. Todos los datos se procesaron con SPSS versión 25.

Se hizo un análisis univariado descriptivo de todas las variables y bivariado descriptivo con la variable sexo.

La variable "perímetro abdominal" presentó un 39 % de los valores perdidos, considerado alto para la imputación, sin afectar la validez.

Para variables cualitativas, se calcularon frecuencias y porcentajes; para cuantitativas, estadísticos descriptivos. Se aplicó la prueba de Kolmogorov-Smirnov, a fin de contrastar la normalidad en muestras grandes.

El análisis de asociación con la variable *sexo* aplicó la prueba de independencia de Chi-cuadrado para variables cualitativas y la prueba U de Mann-Whitney para variables cuantitativas debido a la distribución no normal.

#### Control de sesgos

Aunque el estudio incluyó a todos los que completaron la fase II, podría haber sesgo de selección al no considerar a todos los pacientes de los programas de RC en Bogotá.

Se controló el sesgo de información del digitador verificando aleatoriamente el 5 % de los datos.

Este trabajo cumple con los estándares éticos de la Declaración de Helsinki del 2008, el Decreto 2378 del 2008, las Resoluciones 8430 de 1993, 1995 y 1999 del Ministerio de Salud y Protección Social de Colombia, y la Ley 1581 del 2012 *"Habeas data".* Fue aprobado por el comité de ética en investigación de la institución, mediante resolución Ceifus 479-20 del 31 de marzo del 2020.

## RESULTADOS

### Características poblacionales (Tabla 2)

De 1 737 sujetos, el 61,5% fueron hombres, la edad promedio fue 63 años, el peso promedio 70,8 kg y el índice de masa corporal (IMC) 26,7 kg/m^2^ (preobesidad).

**Tabla 2:** Características generales de la población

n=1737		
Edad (años)		63,1 ± 12,9
Peso (Kg)		70,8 ± 12,4
IMC (kg/m^2^)		26,7 ± 4,0
	(%)	
Sexo hombres		61,5
Hipertensión arterial		66,1
Diabetes mellitus		24,1
Dislipidemia		56,9
Tabaquismo		42,8
Sedentarismo		71,2
Familiares		18,8
Obesidad abdominal		60,5
Preobesidad/obesidad		71,2
Clasificación nutricional	Delgadez	0,6
	Normal	33,9
	Preobesidad	44,0
	Obesidad I	16,1
	Obesidad II	3,2
	Obesidad III	0,2
Fracción de eyección		52,7 ± 12,2
Clasificación FEVI	Reducida	13,9
	Límite	11,8
	Conservada	70,5
Riesgo AACVPR	Bajo	33,4
	Moderado	38,9
	Alto	27,7

El análisis de FRCV destacó al sedentarismo y la preobesidad/obesidad en el 71,2%, seguido de la HTA en el 66,1% y la obesidad abdominal (OA) en el 60,5%.

### Clasificación nutricional

El 44% presentó preobesidad, el 33,9% tuvo peso normal, el 16,1% registró obesidad grado I y el 3,2 % obesidad grado II.

La fracción de eyección (FE) se conservó en el 70,5% (promedio 52%). Alto riesgo según la AACVPR: 27,7%.

### Características y prevalencia por sexo (Tabla 3)

La edad promedio en años fue similar (63,2 hombres y 62,9 mujeres). Hubo una mayor afectación de IMC y perímetro abdominal en las mujeres. La preobesidad/ obesidad afectó al 65,3% de las mujeres y al 57,5% de los hombres. Los hombres demostraron mayor sobrepeso (48,7%vs.38,9%), en las mujeres predominaron la obesidad grado I (20,1 % vs. 14,1%) y grado II (5,2% vs. 2%), y la OA (54,9% vs. 48,6%). No hubo diferencia en obesidad grado III. El tabaquismo fue mayor en hombres. La incidencia fue similar en HTA, DM y DLP. Las mujeres mantuvieron mayor FE.

**Tabla 3:** Análisis de los frcv entre ambos sexos

Características de los sujetos		Hombre n=1068	Mujer n=669	Valor p
Edad (años)		63,2 ± 12,2	62,9 ± 13,9	0,693
Peso (Kg)		74,5 ± 11,6	64,9 ± 11,5	0,000
IMC (kg/m^2^)		26,5 ± 3,6	27,1 ± 4,5	0,007
Riesgo AACVPR	Bajo	26,8	43,9	0,000
	Moderado	41,3	35,1	
	Alto	31,9	20,9	
HTA		65,3	67,6	0,324
DMT2		25,7	21,7	0,059
DLP		57,9	55,3	0,295
Tabaquismo		51,7	28,7	0,000
Familiares		18	20,3	0,223
Preobesidad/obesidad		57,5	65,3	0,001
OA*	Sí	48,6	54,9	0,000
	No	11,9	7,3	
Clasificación nutricional	Delgadez	0,5	0,9	0,000
	Normal	34,5	34,6	
	Preobesidad	48,7	38,9	
	Obesidad I	14,1	20,1	
	Obesidad II	2	5,2	
	Obesidad III	0,3	0,3	
FE (%)		51,2 ±	55,1 ±	0,000
Clasificación FE	Reducida	17,8	9	0,000
	Límite	14,1	9,4	
	Conservada	68,2	81,6	

*Valores perdidos 39 %%.

### Principales diagnósticos de remisión (Tabla 4)

Los principales diagnósticos de remisión fueron el infarto agudo de miocardio (IAM) y la angioplastia.

**Tabla 4:** Diagnósticos más frecuentes de ingreso

Otros diagnósticos	n	%
Infarto agudo de miocardio	741	21,3
Angioplastia	653	18,8
Revascularización quirúrgica	292	8,4
Cardiopatía	285	8,2
Cirugía de reemplazo valvular	164	4,7
Arritmia	126	3,6
Falla cardiaca	90	2,6
Síncope	88	2,5
Otra cirugía cardiaca	65	1,9
Valvulopatía	64	1,8

### Riesgo cardiovascular por sexo (Tabla 5)

Predominio de riesgo moderado y alto en hombres; riesgo bajo similar entre ambos.

**Tabla 5:** Análisis global del nivel de riesgo cardiovascular entre sexos

Nivel de riesgo AACVPR		Bajo		Moderado		Alto		Total	
		n	%	n	%	n	%	n	%
Sexo	Hombre	286	49,3	441	65,2	341	70,9	1068	61,5
	Mujer	294	50,7	235	34,8	140	29,1	669	38,5
Total		580	100	676	100	481	100	1 737	100

## DISCUSIÓN

Pocos estudios nacionales caracterizan a los pacientes en programas de RC. Este estudio destaca por su amplia muestra (n=1 737).

En Floridablanca, Santander, en el 2006 se describen tres estudios en la Fundación Cardiovascular de Colombia. El primero agrupó a 275 pacientes en 16 sesiones de RC, dos veces por semana por ocho semanas, entre marzo y junio del 1999. El segundo, en el 2005, incluyó a 278 pacientes atendidos en 10 años, con un promedio de 17 sesiones; el 17 % hizo menos de 16 sesiones. El tercer estudio, con 75 pacientes de falla cardiaca, duró cinco años y tuvo un promedio de 20 sesiones de RC. Se publicaron datos antropométricos, diagnósticos al ingreso, prevalencia de FRCV y cambios postintervención 13.

En la Clínica Shaio de Bogotá, entre diciembre del 2004 y mayo del 2008, se estudiaron 214 pacientes con diagnóstico de falla cardiaca isquémica, que recibieron 12-24 sesiones de RC, dos veces por semana en promedio. El objetivo fue evaluar el impacto de la RC en la percepción del esfuerzo y rendimiento físico, para lo cual se calcularon los METS a partir del consumo indirecto de oxígeno. El estudio proporcionó datos sobre las características generales y los FRCV predominantes en el grupo 14.

En la Clínica Fray Bartolomé de las Casas en Bogotá, de junio del 2008 a agosto del 2009, se analizaron 14 pacientes con antecedente de enfermedad coronaria que asistieron a 36 sesiones de RC, tres veces por semana por 12 semanas. Cada sesión duró una hora. Aunque el objetivo era evaluar el impacto del programa en la calidad de vida, también proporciona datos relevantes, incluida la prevalencia de FRCV en su población 15.

En el Hospital Militar Central de Bogotá, de junio del 2008 a febrero del 2010, un estudio descriptivo involucró a 173 pacientes en fase II. Se analizaron sus características demográficas, clínicas y el riesgo cardiovascular vinculado al ejercicio, con un promedio de 32 sesiones 16.

En Medellín, una investigación describió características de 77 pacientes en fase II, con seguimiento desde enero del 2006. El programa consistió en 12 a 24 sesiones de ejercicio de una hora, tres veces por semana. Los autores detallaron la estructura del programa de RC, datos demográficos, diagnósticos frecuentes y FRCV prevalentes 17.

En Floridablanca (Santander) se hizo un estudio de cohorte prospectivo, con 72 pacientes mayores de 18 años hospitalizados en unidad coronaria de cuarto nivel entre agosto del 2010 y diciembre del 2011, quienes recibieron RC. Aunque los autores no especificaron la cantidad y la frecuencia de las sesiones, proporcionaron datos sobre características clínicas y el comportamiento de los FRCV con la intervención 18.

Otros trabajos sobre FRCV no están vinculados a intervenciones de RC. Algunos son estudios internacionales multicéntricos que deben considerarse por su amplia cantidad de pacientes y la relevancia de sus datos para nuestro país 2,19-39.

En nuestro estudio, la edad promedio fue 63,1 ± 12,9 años, similar a otros estudios en RC 15-17 y poblaciones no sometidas a este tratamiento 2,40. Infortunadamente, la mayoría de los estudios sobre FRCV en nuestro país incluyó a pacientes más jóvenes, lo cual limitó la comparación. Al igual que en otros estudios 2,26,41, no encontramos diferencias significativas de edades entre sexos.

El 71,2% de nuestra población en el programa de RC es sedentaria y presenta sobrepeso/obesidad en este mismo porcentaje, con predominio del sobrepeso (IMC promedio de 26,7+/-4 kg/m^2^), lo que coincide con otros estudios realizados en el país 2,14,15,17,21,25,39,40,42-46 y destaca la necesidad de reforzar políticas para mejorar la educación nutricional y promover la actividad física en el control del exceso calórico.

En nuestro trabajo, al igual que en otros estudios 2,27, el sobrepeso predominó en mujeres. Sin embargo, en algunos trabajos sobre FRCV las mujeres tuvieron IMC normal 23,47 o incluso obesidad 39. Solo dos estudios informaron un IMC en rango de sobrepeso entre hombres 2,39.

Al igual que otros estudios con poblaciones diferentes 22,31-33,35,36,48-51, observamos un predominio de la OA, con mayor incidencia en mujeres, que podría vincularse a efectos hormonales y a la realización de actividades más sedentarias que los hombres.

En otros estudios nacionales 27,37,51 hallamos mayor porcentaje de pacientes con obesidad grado I en ambos sexos.

Nuestros resultados coinciden con un estudio hecho en Bogotá y conducido por Barragán *et al.* Dicho estudio descriptivo incluyó a 3 316 pacientes, 2 216 de ellos mujeres, evaluados en el programa de Recomendación y Asesoría en Actividad Física (RAFI) durante el 2007 y el 2008. Los autores tomaron medidas antropométricas y aplicaron una encuesta sobre actividad física y antecedentes de ECV en lugares masivos de actividad física. El sobrepeso fue el mayor factor de riesgo (40,3%), seguido del sedentarismo (24,6%). La mayoría de las personas en la adultez temprana y media fueron más sedentarias. Solo el 26 % de las mujeres y el 32% de los hombres se clasificaron como activos físicamente. Este estudio evidenció una diferencia importante en los niveles de actividad física que confirma que los hombres son más activos y proclives a actividades deportivas que las mujeres. Contrariamente a lo habitual, se observó que el sedentarismo predominó entre adultos jóvenes y de edad intermedia (20 a 40 años), atribuido a factores como la pereza, la falta de tiempo y la voluntad individual 24.

La mayoría de los pacientes en nuestro servicio presentan sobrepeso u obesidad y un estilo de vida sedentario, FRCV que contribuyen significativamente a su morbilidad cardiovascular. Afortunadamente, tras los eventos que llevaron a su remisión al programa, muchos demuestran disposición a cambiar su estilo de vida sedentario y hábitos poco saludables. Esto se logra mediante la participación en grupos de RC, junto con el manejo médico, de manera que disminuya el riesgo de nuevos eventos cardiovasculares.

La HTA también destaca como un FRCV que afecta a ambos sexos, con mayor incidencia en mujeres. Alvarado *et al.* informaron una prevalencia de HTA del 57,1%, principalmente en mujeres, con una prevalencia del 54% 52. Esta tendencia se ha observado en otros estudios 36,37,41,53.

Naranjo *et al.* informaron una prevalencia de HTA del 60,7% 18, mientras que el estudio MULATA del 2013, con 2 798 pacientes, estimó una prevalencia del 71% 22. Otros estudios reportaron prevalencias más bajas 13-15,35.

La DM es un FRCV divergente, con estudios cercanos a nuestra prevalencia 2,18,39,43, aunque la mayoría informa prevalencias inferiores. Al revisar entre sexos, las prevalencias de DM en otros estudios también son menores.

En cuanto al tabaquismo, nuestras prevalencias son mayores que la mayoría de los estudios y entre sexos, con predominio entre los hombres.

En orden de importancia, los principales FRCV de nuestra población son: a) sedentarismo, b) preobesidad/obesidad, c) HTA, d) OA, e) DLP, f) tabaquismo, g) DMT2 y h) antecedentes familiares de ECV.

Estudios colombianos recientes señalan que los principales FRCV incluyen DLP del 99,6%, sobrepeso del 80 %, HTA del 78,5%, sedentarismo del 69% y tabaquismo del 27,8 % 17. Hernández señala que la DLP prevalece en un 85,7 %, seguida de la HTA con 57,1 % 15. En Bogotá, Quiroz *et al.* informaron que los FRCV predominantes en su institución son DLP (69,67%), HTA (56%), sedentarismo (42%), obesidad (27,1%), DM (19,2%) y tabaquismo (19,6%). En cuanto a las intervenciones, el 58,8 % se sometió a angioplastia percutánea, el 34,1 % a revascularización quirúrgica y solo el 0,47% requirió marcapasos 14. Báez reportó tres estudios realizados en su institución, de los cuales el primero indicó que los FRCV más comunes son: hipercolesterolemia (64%), estrés (58%), sedentarismo (50%), hipertrigliceridemia (45%) y HTA (45%).

El segundo estudio identificó lo principales FRCV: HTA (58,27%), hipercolesterolemia (66,19%), DM (18,71%), obesidad (12,23%), sedentarismo (44,96%), tabaquismo (34,13%), alcoholismo (18,35%), estrés (75,9%) e hipertrigliceridemia (51,44%). En el tercer estudio, con pacientes en falla cardiaca, los FRCV prevalentes fueron: HTA (57,33%), hipercolesterolemia (12%), DM (24 %), obesidad (16%), sedentarismo (56%), tabaquismo (45.33%), consumo de alcohol (26,67%) y estrés (73,33%) 13.

### Fortalezas y debilidades del estudio

Este estudio, que constituye una primera aproximación a las características de nuestra población, destaca por un significativo número de pacientes en fase II de RC, con respecto a otros estudios en el país con más de una década de antigüedad. Sus resultados, valiosos para la priorización de intervenciones en promoción y prevención, sirven como punto de partida para futuras investigaciones. Sin embargo, tiene limitaciones, siendo sus aportes aplicables principalmente a grupos similares, debido a falta de aleatoriedad en el reclutamiento. La metodología descriptiva y la pérdida de datos en perímetro abdominal (39%) impiden generalizar sobre obesidad abdominal. La falta de análisis sobre variables clínicas adicionales y la imposibilidad de establecer relaciones de causa-efecto son limitantes. Futuros estudios podrían correlacionar el impacto individual y conjunto de los FRCV en la salud de la población. Se destaca la necesidad de nuevos estudios en Colombia para fortalecer las políticas de salud pública, que aborden la educación, la prevención, la detección temprana y la intervención en los FRCV.

El sedentarismo y el sobrepeso/obesidad, son los principales FRCV en nuestra población. Se recomienda fortalecer la orientación nutricional y promover el ejercicio físico para mejorar su control. Estos hallazgos facilitan la definición de metas de intervención y la optimización de estrategias de prevención secundaria en nuestra institución ♠
